# An anti-tumor coup: TIM3 ablation activates the immune arsenal

**DOI:** 10.1038/s41392-021-00757-3

**Published:** 2021-09-27

**Authors:** Kıvanç Görgülü, Derya Kabacaoğlu, Hana Algül

**Affiliations:** Comprehensive Cancer Center Munich at the Klinikum rechts der Isar (CCCMTUM), Technical University of Munich, Munich, Germany

**Keywords:** Cancer microenvironment, Tumour immunology, Tumour immunology, Cancer therapy

In a recent study published in *Nature*, Dixon et al.^1^ showed that TIM3 adjusts “tumor immune temperature” via the involvement of dendritic cells (DCs) with an altered inflammasome activity. Ablation of TIM3 in DCs primarily escalated inflammasome activation and bolstered stem-like CD8^+^ T cells leading to anti-tumor immunity.^1^

In 2002, the discovery of “T cell immunoglobulin and mucin domain-containing protein 3” (TIM3) as an immunoregulatory transmembrane receptor on T-helper cells had led to multiple translational advancements in the field of chronic viral infections, cancer, and other diseases.^2,3^ The translational bravado of TIM3 in the diseases has been evident after being used in several clinical trials. The remarkable common feature of the studies involving TIM3 targeting was overcoming T-cell dysfunction and exhaustion in anti-tumor and anti-viral immunity.^3^ Like in T cells, TIM3 was also present in other cell types such as DCs, myeloid-derived suppressor cells (MDSCs), macrophages, natural killer (NK) cells, and mast cells.^4^ Far more than being expressed in the different immune system players, genetic alteration of TIM3 has also been linked to allergic and autoimmune diseases.^3^ Sakuishi et al. showed that TIM3^+^PD-1^+^ tumor-infiltrating CD8^+^ T lymphocytes (TILs) constitute a significant portion of T cells residing in tumors in preclinical mouse models.^5^ Targeting this overwhelming coupling of TIM3^+^PD-1^+^ depicted drastic tumor regression and reversed these inoperative and severely exhausted CD8^+^ TILs.^5^ Following this study, the strategy of TIM3 ablation has suddenly appeared in clinical trials with the anti-PD-1 combination in solid tumors.

Interestingly, Dixon et al. recently showed that TIM3 hampers anti-tumor immunity of DCs via altering inflammasome and oxidative stress regulation.^1^ Generation of conditional knock-out mice targeting TIM3 in T cells showed that TIM3 influenced tumor growth of immunogenic MC38 colon carcinoma cells (MC38-OVA^dim^) only in modest levels with the involvement of both CD4^+^ and CD8^+^ T cell subtypes. These mild changes steered researchers to reveal the function of TIM3 in myeloid cells, especially DCs, dictating anti-tumor immunity. Single-cell RNA sequencing (sc-RNAseq) analysis of TILs in wild-type mice bearing MC38-OVA^dim^ has illustrated predominant expression of TIM3 on DCs, including DC1s and migratory DCs (migDCs). Tumors have shrunk significantly upon conditional deletion of TIM3 in DCs using *CD11c*^*cre*^ (i.e., *Havcr2*^*cko*^) (Fig. 1). The anti-tumor function of TIM3 deletion in DCs was also observed in non-small-cell lung carcinoma. Moreover, the tumor inhibitory effect of TIM3 deletion was superior in the DCs compared to T cells. Although *Havcr2*^*cko*^ tumors had elevated CD8^+^ T cell infiltration, no noteworthy changes in the levels of cytokines, chemokines, and co-inhibitory and -stimulatory molecules on the DCs were observed.

**Fig. 1:**
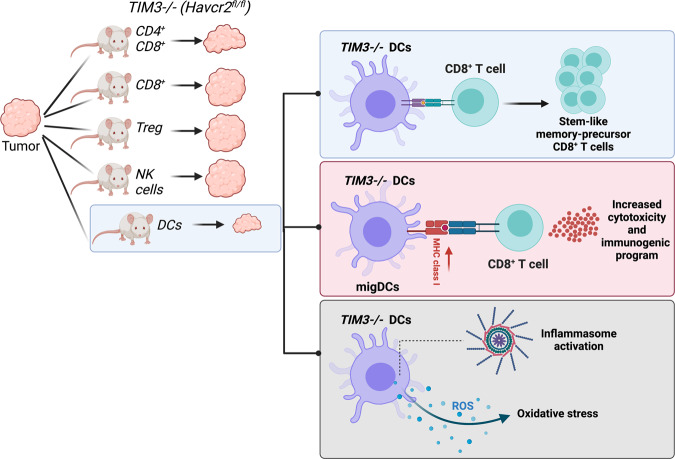
TIM3 adjusts “tumor immune temperature” via dendritic cells (DCs). Among many other immune cells, TIM3 ablation in T-cells shows a mild anti-tumor activity while it reaches the maximum in DCs. TIM-3 ablation increases ROS-induced inflammasome activation, especially in migDC subtypes, subsequently enhancing CD8^+^ T cell cytotoxic activity and stem-like memory precursor formation in immunogenic tumors (created with BioRender.com)

Identifying different cellular clusters in *Havcr2*^*fl/fl*^ *and Havcr2*^*cko*^ via sc-RNAseq delineated the increased proportion of gene signatures belonging to the memory precursor CD8^+^ T cells, presenting stem-like features such as increased proliferative activity to be further exploited via immune checkpoint blockade. In *Havcr2*^*cko*^ mice, they also identified PD-1^+^CD8^+^ TILs expressing more IL-7R, CD5, and CXCR5, pointing to the stem-like features of CD8^+^ TILs. Ablation of TIM3 in DCs promoted the preserved existence of stem-like CD8^+^ T cells, which are therapeutically exploitable (Fig. 1). On the other hand, following TIM3 deletion, sc-RNAseq revealed another subcluster of myeloid cells named migDCs. migDCs showed weakened expressions of the immunoregulatory mediators, including IL-4R, CD200, CD83, and OX-40. Reversing the tumor growth dynamics in *Havcr2*^*fl/fl*^ mice via anti-IL4 treatment has proven that TIM3 ablation enables an antigen-specific anti-tumor immunity (Fig. 1). What caused these effects in CD8^+^ T cells and migDCs following TIM3 loss? With the help of the Waddington OT package, the cellular interactions between the CD8^+^ T cells and the migDCs were exhibited. Provocatively, *Il18-Il18r1* and *Il18-Il18rap* were the ligand-receptor gene pairs showing a significantly increased interaction score. Moreover, *Havcr2*^*cko*^ mice possessed enriched scores for Il18 and *Il1* gene sets in CD8^+^ T cells, allowing researchers to hypothesize an existing link between the loss of TIM3 and inflammasome activation. In parallel, migDCs in *Havcr2*^*cko*^ mice had significant enrichment for the inflammasome activation. Dixon et al. identified an oxidative stress-associated gene signature in migDCs augmenting inflammasome activation in *Havcr2*^*cko*^ mice (Fig. 1). Especially, immunogenic colon carcinoma cells bearing *Havcr2*^*cko*^ mice harbored the increased oxidative stress in DCs. Inhibition of reactive oxygen species via N-acetylcysteine treatment rescued the phenotype of anti-tumor immunity in *Havcr2*^*cko*^ mice. Last, to understand how inflammasome activation precisely controls anti-tumor immune response in tumor-bearing *Havcr2*^*cko*^ mice, the Kuchroo team has utilized three different approaches: (1) caspase-1 inhibition, (2) MCC950 treatment to destroy ASC complex, and (3) blockage of IL-1β/IL-18 axis with the antibodies which was the most vigorous way to lower the tumor temperature.

In the context of cancer therapeutics, targeting TIM3 holds promise as it has already undergone several clinical trials. And yet, from the clinician´s perspective, critical questions remain. In the era of personalized oncology, markers to stratify patients for such combined therapies are required. Do we have to add more markers to the already existing ones (MSI-H, CPS scores)? Is the treatment timeline starting with the conventional chemotherapy always required to deviate from the immunogenic profile of the tumor and the preparedness of it for the TIM3 blockade? If so, are we aware of differences in chemotherapeutic agents interfering with inflammasome activation or inhibition? Should we simultaneously or sequentially combine TIM3 blockade with checkpoint inhibitors in patients eligible for immune therapy? Is TIM3 blockade a way to overcome resistance towards checkpoint inhibitors? Can TIM-3 modulation ex- and in vivo help dendritic cell vaccine engineering along with an increased therapy response? And yet, TIM3 with its mode of action involving the inflammasome, is a refreshing new player in the emerging field of immune therapy and will stimulate new approaches in immune therapy. The future will show how successful and sustainable such clinical studies will be.

## Supplementary information

Pulication License for Figure 1

## References

[1] Dixon KO (2021). TIM-3 restrains anti-tumour immunity by regulating inflammasome activation. Nature.

[2] Monney L (2002). Th1-specific cell surface protein Tim-3 regulates macrophage activation and severity of an autoimmune disease. Nature.

[3] Wolf Y, Anderson AC, Kuchroo VK (2020). TIM3 comes of age as an inhibitory receptor. Nat. Rev. Immunol..

[4] Anderson AC (2007). Promotion of tissue inflammation by the immune receptor Tim-3 expressed on innate immune cells. Science.

[5] Sakuishi K (2010). Targeting Tim-3 and PD-1 pathways to reverse T cell exhaustion and restore anti-tumor immunity. J. Exp. Med..

