# Supplementary material for: An anti-tumor coup: TIM3 ablation activates the immune arsenal
Source: Signal Transduct Target Ther. 2021 Sep 27;6:348. doi: 10.1038/s41392-021-00757-3 (PMC8476541; doi:10.1038/s41392-021-00757-3)
Supplement: Supplementary file 1 — Pulication License for Figure 1 [file 41392_2021_757_MOESM1_ESM.pdf]

639 Queen St. W Suite 401
Toronto ON M5V 2B7 Canada
www.biorender.com

Confirmation of Publication and Licensing Rights

July 21st, 2021
Science Suite Inc.

Subscription:	*Postdoc Plan*
Agreement number:	*GG22QIXSFA*
Journal name:	*Signal Transduction and Targeted Therapy*

To whom this may concern,

This document is to confirm that **Kivanc Görgülü** has been granted a license to use the BioRender content, including icons, templates and other original artwork, appearing in the attached completed graphic pursuant to BioRender's Academic License Terms. This license permits BioRender content to be sublicensed for use in journal publications.

All rights and ownership of BioRender content are reserved by BioRender. All completed graphics must be accompanied by the following citation: "Created with BioRender.com".

BioRender content included in the completed graphic is not licensed for any commercial uses beyond publication in a journal. For any commercial use of this figure, users may, if allowed, recreate it in BioRender under an Industry BioRender Plan.

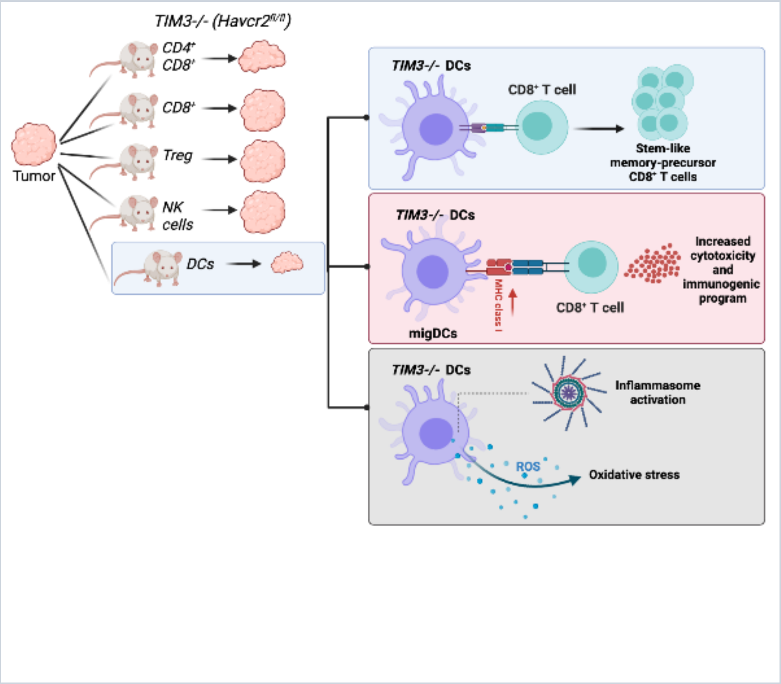

For any questions regarding this document, or other questions about publishing with BioRender refer to our BioRender Publication Guide, or contact BioRender Support at support@biorender.com.
